# Development of the 1.2 T~1.5 T Permanent Magnetic Resonance Imaging Device and Its Application for Mouse Imaging

**DOI:** 10.1155/2015/858694

**Published:** 2015-10-11

**Authors:** Guangxin Wang, Huantong Xie, Shulian Hou, Wei Chen, Qiang Zhao, Shiyu Li

**Affiliations:** ^1^College of Science, Hebei United University, Tangshan 063000, China; ^2^Shanghai Shining Global Science and Education Equipment Co., Ltd., Shanghai 201806, China

## Abstract

By improving the main magnet, gradient, and RF coils design technology, manufacturing methods, and inventing new magnetic resonance imaging (MRI) special alloy, a cost-effective and small animal specific permanent magnet-type three-dimensional magnetic resonance imager was developed. The main magnetic field strength of magnetic resonance imager with independent intellectual property rights is 1.2~1.5 T. To demonstrate its effectiveness and validate the mouse imaging experiments in different directions, we compared the images obtained by small animal specific permanent magnet-type three-dimensional magnetic resonance imager with that obtained by using superconductor magnetic resonance imager for clinical diagnosis.

## 1. Introduction

Magnetic resonance imaging (MRI) can provide nondestructive and high quality CT images that are superior to images yielded by other devices. MRI has been widely used in a series of realms, such as pathology, physiology, and pharmacology. It has brought medical researches into a new era. Due to the complexity of human diseases, many experiments cannot be applied to patients directly. Thus, small animal models are used to simulate the clinical condition of the human body and then applied in medical and clinical researches [1].

Although the animal model MRI in China is still in its infant stage [2], molecular geneticists are doing their best to use animal models to simulate human diseases. In order to develop new drugs and dosage forms, the pharmaceutical industry needs a lot of transgenic or gene-deficient mice as reliable candidates of in vivo detection. Since the transgenic mice are very expensive, traditional monitoring methods such as histomorphology examination are cost-ineffective. Fortunately, MRI technology is becoming mature. Owing to its noninvasion and replicability, MRI technology can help us to save the cost and get encouraging results. Of course, there are great differences between a 20 g mouse and a 70 kg person [3]. When we investigate the mice using the MRI technique which is used for investigations of human body, we will face a dilemma, that is, high cost and poor image quality.

In other countries, scientists have already invented super conductive MRI techniques for the dedicated small animals, which have a dedicated coil with the field strength up to 7.0 T [4]. However, this technique has higher operating costs and it is only used in research without the high cost recovery. Many scientific research institutes and universities cannot afford to operate it. Therefore, there is only a very small amount of such MRI devices in China. Although the technology of permanent magnet device is complex, its cost is low and there are no operating costs. Hence, permanent magnet machine will be the mainstream products for developing countries and regions in a long time. Recently, many research institutes in China began to study the principles of permanent MRI. However, there are only few reports about the application of this technology to manufacture products. Moreover, products with the independent intellectual property are rare. In this paper, we will present the permanent MRI Series (1.2~1.5 T) which is developed by Shanghai Huan Tong Science and Education Equipment Co., Ltd., and Hebei United University.

## 2. Materials and Methods

### 2.1. Materials

**Figure 1:**
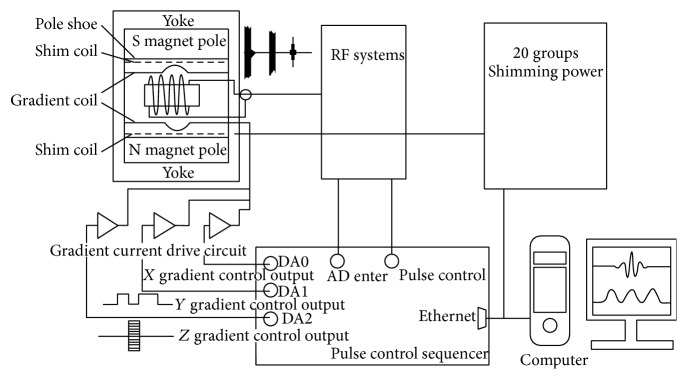
Schematic diagram of MRI structure.

50 N, BH 50 MG Nd-Fe-B steel, gradient coils RF coils, and 12 healthy male mice used in our study were obtained from Shanghai Experimental Animal Center. They are all 4 weeks old with the weight between 17 and 20 grams. The dosage of urethane anesthesia injected into the healthy male mice abdominal is 1 g/kg. The imaging scan was performed 5 minutes later. As a control group, another two Kunming mice (one of them as a spare) with the weight of 20 grams were selected. The abdominal injection of 10% chloral hydrate was performed. Three minutes later, the coronal and sagittal scanning was carried out by using the human body superconducting 1.5 T MRI machine with FOV 100 × 100 mm. The schematic diagram of MRI structure is depicted in Figure 1.

### 2.2. Main Magnet System

As a major component of the MRI device, main magnet system which directly affects the image quality is the key indicators to the entire imaging system. The permanent magnet system used 50 MG energy product Nd-Fe-B magnets. By using numerical analysis and referring to the related references [5–10], we calculated the magnetic pole size depending on the circumstances and obtained the strongest magnetic field with the minimum size.

According to MRI theory, main magnetic field must be uniform. However, the main magnet after that processing cannot meet the requirement. Uniform magnetic fields are mentioned in [8, 9, 11]. Shimming method is firstly proposed by Wenston Anderson in his work [10], in which it gives us the details of the active shim technology. Later on, Dorri et al. described the details of superconducting magnets for passive shimming method [12]. With the development of MRI technology, passive shimming technology research is constantly improved [13, 14] especially after the emergence of permanent magnet devices. Our research focused on passive shimming and combined the use of active and passive shimming methods. We impose *x*, *y*, *z*, *R* ^2^, *x* ^2^-*y* ^2^, *R* ^3^, *R* ^2^ *z*, *y* ^3^, and *xyz*, and other shim coils to build active shimming. Passive shimming is built by the complex processing technology including the calculation through the high-precision calculation, the point by point precision measurement, and subnanometer manual processing technology. We developed measurement mold point by point and choose 2048 measuring point number for measuring accurately and continuously.

Main magnet which is made of sintered Nd-Fe-B permanent magnetic materials has the high magnetic property, but the temperature stability of sintered Nd-Fe-B is poor and it is greatly influenced by environmental temperature [5]. One reason is that the permanent magnet geometry parameters will change with the variation of temperatures. On the other hand, the uniformity of the magnetic field and magnetic field strength are also temperature dependent. Therefore, we cannot get any images. To overcome the disadvantages of poor temperature stability of the permanent magnet, stimulated by the electronic equipment “self-locking” control technology, we developed a self-locking circuit to control the floating of main magnet field strength caused by temperature changes and obtained a steady magnetic field. We have developed the main magnet with independent intellectual property rights, and the main magnetic structures are as follow: the magnet gap is less than 70 mm, the magnetic field strength is 1.2~1.5 T, the diameter of the spherical space is less than 60 mm, the uniformity is 1~0.5 ppm, and the weight is less than 1200 kg.

### 2.3. Gradient Coils and Gradient Field

Shim coils are composed of many directions, namely, *x*, *y*, *z*, *R* ^2^, *x* ^2^-*y* ^2^, *R* ^3^, *R* ^2^ *z*, *y* ^3^, and *xyz*, of which the three coils in *x*, *y*, and *z* directions are used as gradient coils. There is a certain gradient because the magnetic field after the passive shimming is uneven. When the gradient coil magnetic field just offsets the gradient magnetic field of the magnet itself in a certain direction, the gradient coil can be regarded as a shim coil. Therefore, we need to set gradient coils at zero for adjusting the resonant frequency. When the resonance frequencies of different positions are identical and the magnetic field is uniform mostly, we can get the best result of maximum resonance amplitude and resonance signal. According to this principle, we developed adjusting computer software which can be used through the interface buttons to achieve uniform field debugging. We can measure the time stability of the magnetic field as Larmor drift frequency of Hertz per hour. The instrument in this study does not exceed 100 Hz/h. There are a lot of studies of the gradient coil design [15–19] and the permanent magnet-type magnetic field gradient coils [20]. Our study aimed to design self-shielded gradient coils surface with the wavy surface structure. And the new device has the advantages of low eddy current, high efficiency, and high linearity.

### 2.4. RF Coil for Transmitting and Receiving a Signal

**Figure 2:**
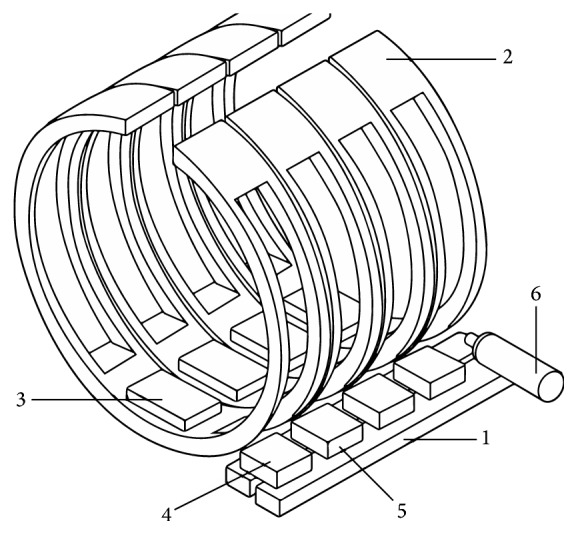
A schematic diagram of the RF coil structure, “1”: backbone transmission wires, “2”: four segments of the coil, “3”: resonant capacitor, “4”: decoupling capacitor, “5”: coupling capacitor, and “6”: wiring as input-output interface with the circuit connection.

**Figure 3:**
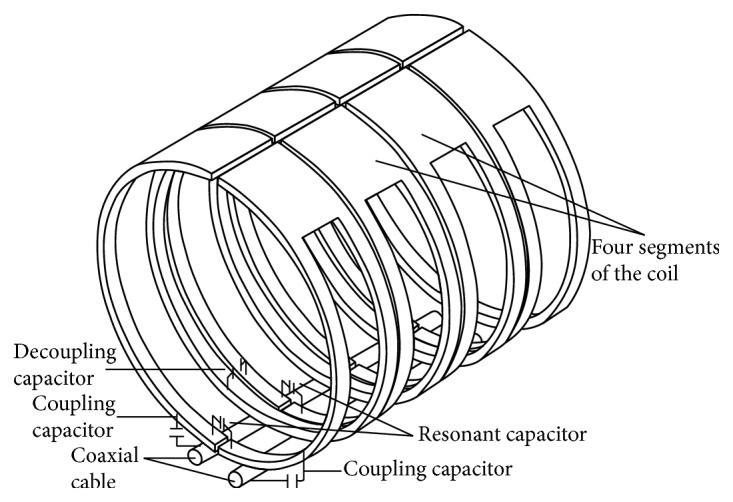
RF coil capacitor connection mode.

RF coil (RF) is used to transmit and receive signal. In order to get the high quality image, the transmitting coil requires not only a faster and higher efficiency of energy conversion but also a uniform RF field that can guarantee the uniform intensity of biological samples. It is essential for receiving coil to have a higher detection sensitivity and ability to reflect the tissue adjacent to the appropriate minor differences so that it can guarantee sufficient signal-to-noise ratio. Only images that can reflect minor differences within the same organization and different organizations in terms of gray difference with distinguishable resolutions are useful. But it cannot simultaneously satisfy the transmitting coil high uniformity and high sensitivity and high signal-to-noise ratio of the receiving coil. According to Biot-Savart law, the magnetic field strength is inversely proportional to the square of the distance. The smaller the distance between the coil and the sample is, the stronger the magnetic field is, and the poorer the uniformity of the RF is. If we divide it into two groups of coils, they will be coupled with each other and lead to the reduction of signal-to-noise ratio. Therefore, the optimization of RF coil has been one of the hot issues in the research of MRI [21, 22]. The shape of the coil has been considered to be the most important factor affecting the coil quality [23]. Our designed RF coil absorbs the advantage which the saddle shaped coil can provide the uniform RF field in the vertical direction of main magnetic field. It also absorbs the advantage of high sensitivity and uniformity field from the solenoid coil. Theoretically, the transmission and acquisition are two groups of coils. In order to save valuable space of main magnetic field, they are integrated together. The current direction is controlled through the circuit, so that the deformation is equivalent to the saddle shaped coil signal transmission along the *x*-axis. The received signal is equivalent to the solenoid coil deformation along the *z*-axis. Thus, the transmitting and receiving signals are orthogonal and without coupling interferences. In order to eliminate the nonuniformity of the launch site, we employed the weighted correction method. By using our revised computer software [24], we obtained the brightness distribution function of the water mode image and then divided it by the function of the actual image. By setting a RF coil into 4 groups of coils in parallel as shown in Figures 2 and 3, we can reduce resistance and loss and increase the magnetic field uniformity and the sensitivity of the coils.

RF coil is fixed on the main magnet cavity, which produces hard and soft pulses. The frequency of RF coil has the adjustment range of 0 to 70 MHz with adjustment accuracy in the step of 0.01 Hz. Since the DDS technology is used, the frequency will have a high stability (10^−8^).

### 2.5. Mouse Imaging

The instrument parameters are as follows: (1) the main magnetic field strength is 1.5 T, shimming volume diameter 35 mm ball, SE weighted T1 sequence, TR = 100 ms, TE = 15.5 ms, and FOV 35 × 35 mm; (2) the main magnetic field strength is 1.2 T, shimming volume diameter 60 mm ball, SE weighted T1 sequence, TR = 100 ms, TE = 15.5 ms, and FOV 50 × 60 mm. Number of excitations NEX = 2.

Cross-sectional scanning is as follows: (*x* and *y* directions phase codes, *z* direction frequency code) data matrix is 32 × 512 × 256 and image matrix is 1024 × 1024. And coronal section scanning is as follows: data matrix is 32 × 512 × 256 and image matrix is 512 × 512. We completed T1-weighted images as scan mode 3D, the sinc RF pulse. The 12 healthy male mice we used in this study were obtained from Shanghai Experimental Animal Center. They are 4 weeks old with the weight between 17 and 20 grams. The dosage of urethane anesthesia injected into the healthy male mice abdominal is 1 g/kg. The imaging scan was performed 5 minutes later. As a control group, another two Kunming mice (one of them as a spare) with the weight of 20 grams were selected. The abdominal injection of 10% chloral hydrate was performed. Three minutes later, the coronal and sagittal scanning was carried out by using the human body superconducting 1.5 T MRI machine with FOV 100 × 100 mm.

### 2.6. Phantom Making

Two water plexiglass cylindrical molds containing 0.3% aqueous solution were made. One is with a diameter of 22 mm and height of 30 mm, the other is with a diameter of 48 mm and height of 60 mm.

## 3. Results

The self-developed instrument is a device which is very complex, having not only the magnetic system of high quality but also a software system of high quality, research and development of computer integration software, and other supporting devices. The software includes the computer control system and the order of accuracy control, release, and storage and accepts a programmable pulse sequence generator, pulse sequence control, image data acquisition and control, data processing system, and signal visualization system. We also successfully developed other types of equipment such as the preamplifier, power amplifier, switch circuit, 3D display software, and 3D NMR integration software [24]. A series of 1.2 T~1.5 T MRI instrument was developed. Select the two instruments for imaging experiments: in the first instrument the magnetic pole gap is 42 mm, weight 400 kg, the magnetic field strength 1.5 T, and shimming 35 mm diameter spherical volume of space, and in the second instrument the magnetic pole gap is 70 mm, weight 1200 kg, the magnetic field strength 1.2 T, and shimming 60 mm diameter spherical volume of space.

### 3.1. Phantom Imaging

**Figure 4:**
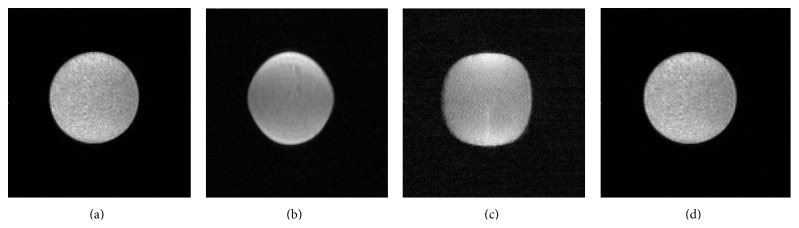
Comparison of axial T1-weighted phantom images with different linearity of gradient field: (a) nonlinearity 0.3%; (b) nonlinearity 5%; (c) nonlinearity 10%; (d) RF coil phantom image 48 mm aperture, 1.2 T.

In order to determine the gradient field suitable linearity of the permanent magnetic field, phantom image suitable gradient filed was obtained through computer simulation [24] and phantoms adjustments.  Figure 4 shows phantom images: (a) nonlinearity 0.3%, (b) nonlinearity 5%, and (c) nonlinearity 10%.

We further improved the uniformity of the RF field through the water mask weighted imaging correction (RF coil research and development (R & D)). In order to ensure the quality of image, the brightness distribution of computer software is divided by the field. The final phantom image is shown in Figure 4(d). As shown in Figure 4(d), a homogeneous solution state without distortion was well demonstrated.

### 3.2. Mice Image

**Figure 5:**
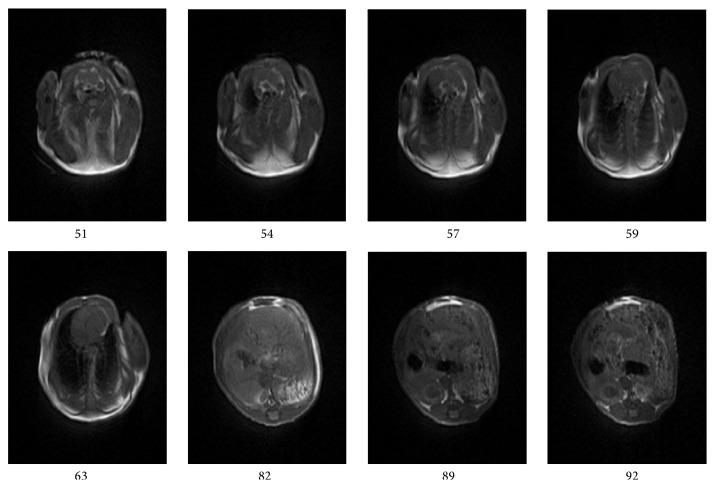
The eight axial T1-weighted mouse images, thoracic abdominal segment levels. Internal organs were clearly observed (1.5 T, 35 mm).

**Figure 6:**
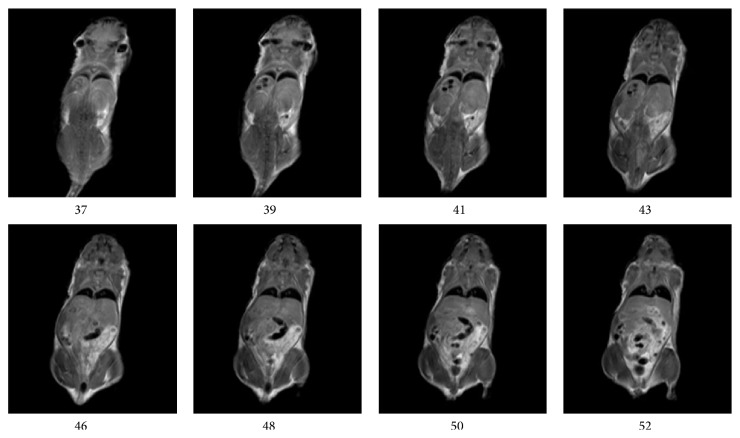
The 8 coronal T1-weighted images, all clear images from head to tail were clearly obtained (1.2 T, 60 mm).

In this study, the three-dimensional imaging technology was employed. Therefore, we can achieve continuous acquisition of a magnetic resonance signal, make the slices closely co-coordinated, and overcome the problem of signal leakage between slices. In order to stimulate the entire slices of blocks at the same time, we put RF pulse width as 50 KHz, which can greatly shorten the pulse duration and reduce the TE, which further reduced the relaxation of the signal loss and yielded a higher signal-to-noise ratio (SNR). Thus, we can get high quality images in the moderate intensity field (1.2~1.5 T). We obtained similar images by using different mice and different test time. This result indicates that the performance of the developed instrument is repeatable and stable. In each test, the number of slices is 128. The chest and abdomen imaging is obtained by cross-sectional scans (imager: 1.5 T, 35 mm) with the slice thickness of 0.3 mm and is shown in Figure 5. Figure 5 shows the axial T1-weighted mouse images. Although they are not as clear as the high-field picture, at the level from the chest to abdomen, the stomach, the large intestine, muscles, jejunum, cecum, heart, vertebra, and spinal cord could all be well observed. The body image is obtained by coronal scans (imager: 1.2 T, 60 mm) with the slice thickness of 0.4 mm and is shown in Figure 6. Figure 6 shows 8 coronal T1-weighted images, all of which are relatively clear images from the head to the tail of mouse. In addition, it is possible to distinguish clearly anatomical structures including the muscles, brain, ear, kidney, liver, stomach, jejunum, cecum, and internal organs and, overall, the entire body of the mouse was seen on one image homogeneously, and the digital under the picture indicates the order to be cut.

## 4. Discussion

### 4.1. Field System

In this study, the development of the magnetic field system is based on previous micromagnetic resonance imaging teaching instrument. The magnetic system is the most important part of MRI, especially the main magnetic field intensity and uniformity. For the permanent magnet type, the improvement of the core magnetic field strength depends on the techniques of alloy. For shimming, in the same magnetic field uniformity, the stronger the main magnetic field is, the greater the bias of magnetic field is. The passive shimming is more detailed. The active shimming of 60 mm aperture may achieve the third-order shimming by applying 20 groups shim coils; therefore, the nonlinearity of the magnetic field can attain 0.8 ppm, and the image quality has been greatly improved.

Since the size of mouse is small, in order to get clear images showing details of the internal organization, we must choose thin slice (0.5 mm or less). Therefore, the requirement of the gradient field linearity should be much higher than that proposed in the literature (<5%) [7]. In this study, the linearity is 0.3%. Since the imaging quality is affected by the linearity of the gradient filed, the methods of how to determine the linearity of the gradient filed have been discussed in the past [25]. However, when the primary magnetic field strength is 1.5 T, the presence of the pole shoe and the yoke of ferromagnetic material can lead to the dramatic increase of eddy current. Therefore, the coil can not solve the self-shielding properties. By improving the pole shoe blocking-up magnetic induction, the eddy currents control is in the acceptable range.

Instead of the conversion ratio of signal to noise, previous studies paid more attention to the coil *Q* value. However, we realize that the most important is the coil sensitivity. The higher the sensitivity, the better the signal-to-noise ratio, which is the basis of yielding the high resolution images. However, the sensitivity is determined by the induction coupling degree and resistance coupling efficiency. Impedance coupling efficiency comprises a coil resistance, sample conductivity decay. Since the conductivity of small animals is higher than human beings, the detected signal will be dramatically decrease. Absorption of phased array coil advantage [26], with small coils which are combined into a large coil, on one hand reduces the resistance of the coil to reduce the loss and on the other hand is decomposed into multiple small coils which are combined together, and the received signals overlap with each other to reduce reflection loss and improve the sensitivity of the coil. The invention of the connection of alloy for high conductive MRI [27] for coil production, between the various components, further reduces the loss. Coil using single channel retains the advantages of low noise single coil, avoiding the multichannel acquisition for the elimination of small coil coupling and acquire complex matching circuit. Our equipment is different from that of conventional RF coil (keel single channel RF coil) [28]. Our equipment retains not only the phased array coil and high filling rate and high sensitivity, but also the single channel properties of body coil as shown in Figure 3. The main characteristics and advantages of the novel equipment are as follows: the load coil *Q* value decreased slightly; the signal sensitivity is two times higher than other signal interfaces; single input and output interface features, and coils in the equipment can mutually inductance and decouple.

### 4.2. Comparison with Human Body's Superconducting Instrument

**Figure 7:**
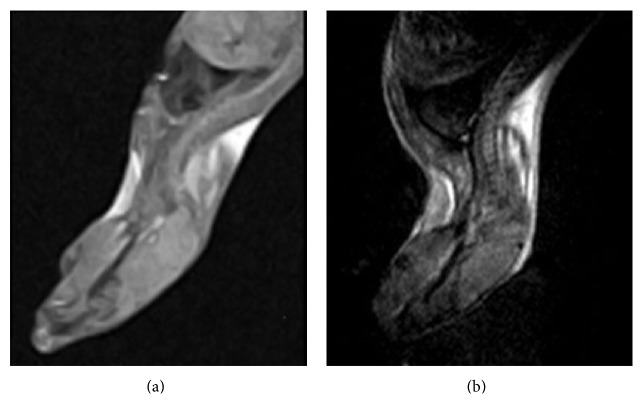
Comparison with human body's superconducting instrument. The left figure is obtained using human device, and the right is obtained in this study.

**Figure 8:**
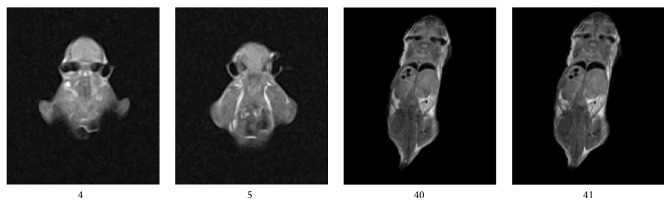
Comparing the slice jump between the fourth and fifth slices with thickness of 3 mm and the fortieth and forty-first slices with thickness of 0.4 mm.

We used the superconducting 1.5 T device with the data matrix 256 × 256, TI = 750.0 ms, TR = 2118.8 ms, and TE = 10.7 ms. We selected minimal wrist surface coil in our hospital and chose the thinnest slice of 3 mm. The T1-weighted images are shown in the left of Figure 7. And the image on the right of Figure 7 is the image obtained by the instrument used in our studies. Because of different position in the machine, mouse is supine in the human body's machine. But in this study, mice were hanging upside down in a magnetic field. The sagittal planes have made a different width under the action of the gravity. Thus, we can only take roughly the same level compared with the position. Figure 7 shows that the human body's MRI device has a very good signal-to-noise ratio. But it cannot distinguish the details. Therefore, if the human body's device is with no special configuration, it cannot be directly used for mouse imaging. On the other hand, wrist coil is too large for a mouse, but the coil is too expensive, and the coils dedicated to a small animal in general hospital generally cannot be configured. The body's tissues and organs are larger, so we generally select the thinnest slice of 3 mm. However, there still exist the following three problems. The 3 mm is too thick mice. There are imaging slice gaps for 2D imaging. The change of imaging slice gaps is so fast that it is inconvenient for further researches. Therefore, 3D imaging can be a very good solution to this problem. In this study, the slice of cutting mouse head is about 60 slices.  Figure 8 shows the fourth and fifth slices with thickness of 3 mm and the fortieth and forty-first slices with thickness of 0.4 mm, respectively. Human device is not easy to be used directly on mice, and the general research institutes cannot afford the cost. Therefore, the development of permanent significance of small animal MRI is urgent.

For the convenience of the vast majority of experimental scientists, we developed a suite of software package to help users to get the desired results. The software package includes the computer control system and the order of accuracy control, release, and storage and accepts a programmable pulse sequence generator, pulse sequence control, image data acquisition and control, data processing system, signal visualization system, the preamplifier, power amplifier, switch circuit, and 3D display software [24].

## 5. Summary

In summary, the study of 1.2~1.5 T permanent MRI obtained a preliminary success. We can see that the tissues and organs of the head or abdomen and the body of the mouse can be clearly distinguished (Figures 6 and 7). But for the levels close to the initial or end of the slice, there is high noise so that we cannot observe the organizational structure. This is due to the fact that our uniform field space is spherical with a diameter of 35 mm (1.5 T) and 60 mm (1.2 T) space but not a cylindrical space. In addition, the SNR decreases along with the increase of RF coil size. The best performance can be obtained at the spiral coil intermediate position, where the SNR achieved the highest value. Because it is three-dimensional imaging, we can adjust the position of mouse in the main magnetic field to the best region of interest. But there are still some shortcomings. Firstly, there is only T1-weighted for the relatively mature sequence, and T2-weight images are not stable enough in vivo. Since T2-weighted is widely used in disease analysis, we will develop more imaging sequence in the future. Secondly, we will improve mouse fixtures and unify the mouse body so that the image will be more comparable.
